# Royal Westminster Ophthalmic Hospital

**Published:** 1829-10

**Authors:** 


### ROYAL WESTMINSTER OPHTHALMIC HOSPITAL.

The readiness with which Mr. Guthrie gives a trial to every remedy which is supposed, by any adequate judge, to possess peculiar powers, is as laudable as the attention paid to the cases whilst under treatment. They are delivered over to the various students in attendance, whose names are attached to the cases, and are the best vouchers for their authenticity. From 150 to 200 persons are seen every Tuesday and Thursday morning, each being called up according to a numerical ticket given to them on their arrival. Of late, the Oleum Terebinthinæ has been the great object of attention, and upwards of thirty cases have been treated, nearly all with complete success. We have selected the following as illustrative of its powers in various kinds of inflammation of the more internal parts of the eye, to which its use has been principally restricted. It will be seen that in some it supersedes the use of mercury, which in many constitutions it is most desirable to abstain from, and this even in pure cases of syphilitic iritis. In the rheumatic iritis it is certainly a most efficient remedy, and in many other more anomalous affections it seems decidedly advantageous. It is administered in drachm doses, with mucilage and any medium which may make it most agreeable, and is continued until its action upon the kidneys and bladder becomes evident: indeed, few patients seem to benefit by its use until the scalding of the water, and the increased desire to pass it, become troublesome. When these symptoms are too severe, diluents, such as linseed-tea, &c. are had recourse to, with gentle purgatives, opiates, warm bath, &c. In no one instance has any permanent inconvenience occurred from its use.

Case I.—George Kemmis, æt. fifty-one, a tall athletic man, in March began to see double, accompanied by uneasiness in the left eye, supposed to be from the east wind: for this he was bled, cupped, and purged, with advantage. The middle of May he suffered an attack of rheumatic inflammation of the internal parts of the same eye, for which he took bark, soda, colchicum, and mercury, with comparatively little advantage.

On the 1st of July he was directed to take the Ol. Terebinth. ʒi. three times a day. It was continued at that dose for three weeks, and removed the pains in the forehead, side of the head, and eye, which were never completely relieved by the other means employed. He lost also, under this treatment, the double vision which he had suffered from since March. The turpentine was continued for three weeks more, twice a day; and occasionally since, when he finds any inconvenience.

The pupil is natural in size, but slightly irregular, adhering to the capsule of the lens; an effect which generally takes place after rheumatic inflammation, when not rapidly cured, rendering it at all times an infinitely more dangerous disease than when the cause is syphilitic or otherwise.

In this case, one of pure rheumatic inflammation of the choroid coat and iris, the turpentine exhibited greater powers than any of the other remedies employed; and the same thing has been observed in other instances of a similar character.

Case II.—R. H. Hill, æt. twenty-nine years. About four years ago he lost the sight of his left eye from inflammation, over which a broad nebula had spread itself. Before seeing Mr. Guthrie, the eye had been much weakened by two large ulcers. The inflammation of the eye was general, and the edges of the pupil were irregular. Sixteen ounces of blood were taken from the right temple by cupping, and six grains of calomel and a black draught administered. The patient's bowels not yielding, the dose was given for three successive days. Neither the cupping nor medicine appeared to have the slightest effect; for, on the 10th of August, two fresh ulcers had formed immediately upon the edge of the transparent cornea, causing violent inflammation, great tension of the lids, and an intolerance of light.

On the 11th, an effusion of lymph was observed on the edge of the iris. The patient was again cupped to twelve ounces, and a drachm of Ol. Terebinthinæ administered three times a day. The cupping produced no effect: on the contrary, the eye was fast getting worse. On the 16th the patient was again cupped, but, as before, without receiving any benefit.

On the 18th, the turpentine, which had been regularly taken, began to affect the kidneys and the whole course of the urethra. Blood was freely voided per urethram; and a distressing scalding sensation was felt down its whole course. Besides these effects of the Ol. Terebinthinæ, there were others which are worth remarking. The oil, when taken, produced a warmth in the stomach, which was quickly succeeded by nausea, stupor, and excessive languor and depression of spirits: these symptoms, in their turn, were succeeded by heartburn, and great coldness of the extremities; the patient's sensation was as if the blood of the head and extremities were congregating about the heart.

From the time the turpentine thus affected the patient, his eye became evidently better. The secretion, which had been very great and scalding, now became cool; no pain was felt at the temple, and light was no longer intolerable. On the 22d, the ulcer and inflammation had disappeared; the inflammation of the iris, and effusion of lymph which it had caused, had both vanished. The patient was pronounced to be cured, and permitted to return into the country.

It is stated above that the patient had lost the sight of his left eye: this eye has improved gradually with the other, under the course of turpentine. The mist is not so thick, and appears to be going away.

Case III.—John Toury, æt. twenty-five, admitted August 6th, 1829, with pustular inflammation, of three weeks' standing. A pustule, of the size of a pin's head, is situated on the inner and lower edge of the cornea; the conjunctiva is greatly inflamed, especially around the pustule; there is great flow of tears; the lids adhere in the morning; iris slightly changed in colour. He complains of a little pain in the eye.—Appl. Unguent. Argent. Nitrat.

August 8th.—The inflammation is a little diminished.—Guttæ Argent. Nit.

9th.—Much worse. The conjunctiva is considerably more inflamed; he complains of great pain over his brow.—Appl. Ung. Argent. Nitrat. Cap. Pulv. Jalapæ comp. ℈iss. statim. Cucurb. cum ferro applicent. ad temp. ad ℥xiv.

13th.—To be cupped from the temples again.

15th.—He feels much worse, and complains of great pain round the orbit, with intolerance of light. The iris is rather darker than natural, and acts irregularly; the inflammation of the conjunctiva amounting almost to chemosis, and the state of the sclerotic coat, therefore, cannot be seen.—℞. Vin. Colchici f ℨi.; Pulv. Ipecac. comp. gr. x.; Aquæ ℥iss. h. s. s.

16th.—The pain is much diminished, and he can bear the light much better; the vessels of the conjunctiva not quite so much distended as yesterday; the pupil is a little irregular.—Rep. Haust. Colchici h. s.

17th.—He has no pain now, except on exposure to light; the pustule on the margin of the cornea is advancing; the iris remains of a darker colour, and the pupil is of an oval shape.—Repet. Haust. Colchici h. s. s. Guttæ Vini Opii. Applicatur Emplast. Bellad. tempori.

18th.—He has no pain whatever in the eye; the vascularity of the conjunctiva is diminished.—Guttæ Argent. Nitr.

19th.—The conjunctiva is much more distended, and the pain much increased.—Rep. Guttæ Opii. Omitte Guttæ Arg. Nitr.

20th.—He complains still of great pain in the head, which is not so severe as yesterday. The anterior chamber is rather muddy. —App. Ung. Arg. Nitr. To be cupped from the temples to twelve ounces.

22d.—The iris is more discoloured and contracted; the turgid state of the conjunctiva much the same. He sees much better, the iris being now principally affected, (not, however, apparently from any venereal disease.)—He was ordered to take Ol. Terebin. ʒi. ter die.

24th.—The conjunctiva is evidently less injected; the iris of a lighter colour; the muddiness of the anterior chamber rather increased, and he does not see so well. He complains of little pain. The pustule has burst, and left a deep ulcer, implicating the lower part of the inner margin of the cornea. The medicine has produced no unpleasant sensation about the urinary organs.—Repet. Ol. Terebinth. ʒi. ter quotidie.

25th.—Pain in the head gone; the iris is less discoloured; the anterior chamber rather clearer, and he sees much better.—Cont. Ol. Terebinth. ʒi. ter die. Capiat. Magnes. Sulph. ℥i. mane.

29th.—Has taken the medicine regularly since the 25th. The vascularity of the conjunctiva is much diminished, and a zone of a pink colour can now be distinctly seen round the margin of the cornea; the pupil is regular, but contracted, the colour of the iris much the same. His sight improves daily; he has no pain in the head, but complains of slight strangury.—Cont. Ol. Terebinth. ʒi. ter die.

September 1st.—The inflammation of the conjunctiva nearly gone; the zone round the cornea is of a much darker colour, and very distinct; the pupil is a little irregular; the ulcer on the margin of the cornea increased in size. He has no strangury.—Cont. Ol. Terebinth. ʒiss. ter die.

6th.—Has been confined at home by severe pains of the abdomen, and sickness, on account of which he has not taken the Ol. Terebinth. since the 1st. He makes water more frequently, but without scalding: the eye is much improved; the zone less distinct, and of a paler colour; the pupil is rather more dilated, but still a little irregular; the iris is of a lighter colour.—℞. Ol. Terebinth. ʒiss.; Tr. Opii gtt. x.; Mucil. Acaciæ ℥ss.; Aquæ ʒvij. M. fiat haustus ter die. Capiat Ol. Ricini mane. Guttæ Argent. Nitr.

9th.—The pupil rather more irregular; the iris is now of its natural colour; the sclerotic inflammation nearly disappeared, and the ulcer of the cornea is less. He sees as well as ever he did.—Cont. Ol. Terebinth. ʒi. ter die. Rep. Guttæ Argent. Nitr.

11th.—The ulcer is filling up. There is still a little vascularity of the sclerotica. He complains of rheumatic pains over his temples and forehead; for which he was ordered Pulv. Ipecac. comp. gr. x. hâc et crastin. nocte. Pil. Colocynth. cum Calomel mane sumend. Omitte Ol. Terebinth.

15th.—The iris is a little darker; the pupil is still contracted, but regular, and the sclerotic is very slightly injected. The ulcer is nearly healed; the rheumatic pains much relieved.—Guttæ Argent. Nitr.

22d.—The ulcer has not quite healed, but he is well in all other respects.—Unguent. Arg. Nitr.

Case reported by Mr. Weight.

